# Labour market participation after sickness absence due to cancer: a dynamic cohort study in Catalonia (Spain)

**DOI:** 10.1186/s12889-023-17321-z

**Published:** 2023-12-11

**Authors:** Amaya Ayala-Garcia, Fernando G. Benavides, Laura Serra

**Affiliations:** 1 Center for Research in Occupational Health (CiSAL), Universitat Pompeu Fabra, PRBB Building, Dr. Aiguader, 88, 08003 Barcelona, Spain; 2 CIBER of Epidemiology and Public Health (CIBERESP), Madrid, Spain; 3 IMIM – Parc Salut Mar, Barcelona, Spain; 4 Research Group On Statistics, Econometrics and Health (GRECS), University of Girona, Girona, Spain

**Keywords:** Sickness absence, Cancer, Return to work, Permanent disability, Retirement, Longitudinal study, Sequence analysis

## Abstract

**Background:**

The consequences of cancer on working until retirement age remain unclear. This study aimed to analyse working life considering all possible labour market states in a sample of workers after sickness absence (SA) due to cancer and to compare their working life paths to those of a sample of workers without SA and with an SA due to other diseases.

**Methods:**

This was a retrospective dynamic cohort study among social security affiliates in Catalonia from 2012–2018. Cases consisted of workers with an SA due to cancer between 2012–2015 (*N* = 516) and were individually age- and sex-matched with those of affiliates with an SA due to other diagnoses and workers without an SA. All workers (*N* = 1,548, 56% women) were followed up from entry into the cohort until the end of 2018 to characterise nine possible weekly labour states. Sequence analysis, optimal matching, and multinomial logistic regression were used to identify and assess the probability of future labour market participation patterns (LMPPs). All analyses were stratified by sex.

**Results:**

Compared with workers with an SA due to cancer, male workers with no SA and SA due to other causes showed a lower probability of being in the LMPP of death (aRRR 0.02, 95% CI: 0.00‒0.16; aRRR 0.17, 95% CI: 0.06‒0.46, respectively) and, among women, a lower probability of permanent disability and death (aRRR 0.24, 95% CI: 0.10‒0.57; aRRR 0.39, 95% CI: 0.19‒0.83, respectively). Compared to workers with SA due to cancer, the risk of early retirement was lower among workers with no SA (women, aRRR 0.60, 95% CI: 0.22‒1.65; men, aRRR 0.64, 95% CI: 0.27‒1.52), although these results were not statistically significant.

**Conclusions:**

Workplaces, many of which have policies common to all diagnoses, should be modified to the needs of cancer survivors to prevent an increasing frequency of early retirement and permanent disability when possible. Future studies should assess the impact of cancer on premature exit from the labour market among survivors, depending on cancer localisation and type of treatment.

**Supplementary Information:**

The online version contains supplementary material available at 10.1186/s12889-023-17321-z.

## Introduction

In 2020, 385 new cases of cancer per 100,000 people aged 20–64 years were diagnosed in Europe [1]. This figure represents nearly 50% of the total number of new cancer diagnoses [2], and the average 5-year survival of malignant neoplasms considering all ages has reached almost 54% [3]. A recent report predicted an extension of working life, with a 10% increase in the participation rate among workers aged between 64 and 74 years by 2070 in the EU-27 [4]. Therefore, an increase in the number of people diagnosed with cancer while working is expected.

Currently, most cancer survivors take a sickness absence (SA) during their treatment to overcome the acute stage of the disease [5] with the intention of returning to work when treatment ends. SA due to cancer tends to be longer than SA due to other diagnoses [6]. After the treatment stage, some cancer survivors face adverse effects that can persist for prolonged periods or become chronic due to the treatment or the severity of the disease itself [7]. When these symptoms impair work performance, cancer survivors may decide to ask for permanent disability (PD) benefits from the social security system. The process of returning to work is also affected by other factors. On the one hand, sociodemographic factors such as age, sex, education level and family support influence the ability to continue work after cancer. A recent study showed that female cancer survivors dropped out of work more often than controls of either sex, were less likely to work full-time than males, and increased their participation in short part-time work more often than male survivors [8]. On the other hand, work and employment conditions, job strain, physical job demands, type of job, support at the workplace, type of contract and previous periods of unemployment are also influential factors [9–11]. However, most cancer survivors attempt to return to work (RTW) after the first year or a maximum of 2 years after diagnosis, right after the SA ends [12].

Studies on long-term working life, including all possible working paths after cancer, are scarce. Most studies have investigated the probabilities of future labour outcomes individually, which obviates transitions between these states until retirement. Characterising the working paths of cancer survivors may shed light on how surviving the disease and subsequent career decisions may interact in the long term. In a previous study on employment trajectories after an SA due to cancer, we showed that cancer survivors are less likely to accumulate days of employment in the long term [13]. On the one hand, these changes in survivors’ working life may be driven by personal decisions due to health or financial status or a desire to modify career paths after a reassessment of life priorities [14]. Hence, cancer survivors may decide to work fewer hours than before the SA, take time off from work for prolonged periods, and experience cancer-associated long- or short-term job loss with or without unemployment benefits [15]. In addition, some survivors choose to change their retirement plan by retiring early or before they planned to [15]. On the other hand, these decisions could also be driven by labour market or workplace demands. For example, some survivors may not be able to perform a high-strain job due to long-term side effects impairing their ability to work [16], and there may be a lack of appropriate jobs for their new health status. These decisions may take place at different points after RTW and combine differently depending on opportunities in the labour market and workplace factors. The negative impact of cancer on working life could increase inequalities that could and should be addressed to lessen financial and psychological consequences.

**Fig. 1:**
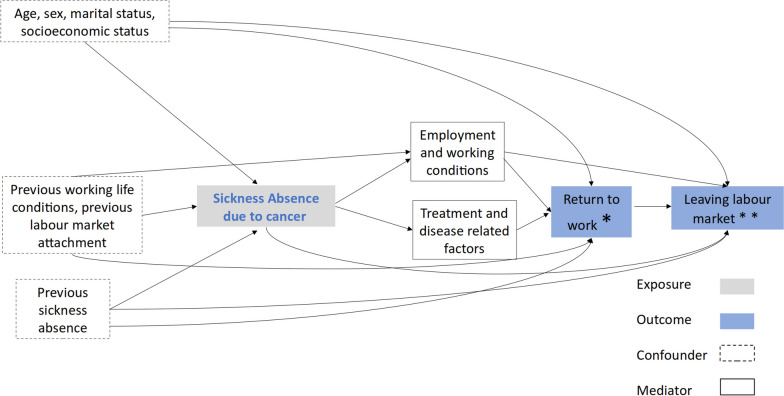
Directed acyclic graph (DAG) of the relationship between a sickness absence due to cancer and future working life. *Categories of the variable “Return to work t1”: temporary employment, unemployment, permanent employment; ** Categories of the variable “Return to work t2”: permanent disability, early retirement, retirement, inactivity and premature death

We hypothesised that cancer survivors would have more unstable career paths after returning to working activity and have a higher probability of prematurely leaving the labour market than people with other diseases or the general working population (Fig. 1).

The objective of this study was to analyse future labour participation trajectories considering eight possible labour market states (temporary and permanent employment, unemployment, inactivity, permanent disability, early retirement, ordinary retirement and premature death) in a sample of workers affiliated with social security after an SA due to cancer and to compare their working life paths to those of a sample of workers without SA and to those of workers with an SA due to other diseases.

## Methods

This study was based on the Spanish WORKing life social security (WORKss) cohort [17]. Briefly, the WORKss cohort is based on the Continuous Working Life Sample (CWLS), which has taken an annual random representative sample of 4% (approximately one million workers) of affiliates of the Spanish social security system since 2004. This database contains the full employment history of each affiliate, including information such as occupational category, the company’s economic activity, employment conditions (e.g., type of contract, income, and working time), social benefits (e.g., unemployment, permanent disability [PD], and retirement), other work-related variables (e.g., company ownership and size), and date of death.

In addition, in this study, we linked the database of the Catalan Institute for Medical and Health Evaluations (ICAM by its acronym in Catalan), from which we obtained information on SA records between 2012 and 2015, including the medical diagnosis of the episode coded according to the 10th Revision of the International Classification of Diseases (ICD-10), as well as the start and end date [18] of workers affiliated with the Spanish social security system in Catalonia, who were also part of the Spanish WORKss cohort.

We performed a retrospective dynamic cohort study among 1,548 (675 men and 873 women) salaried workers living in Catalonia. For the study population, the inclusion criteria were affiliation with the general scheme of social security and residence in Catalonia between 2012 and 2015 (observational period for SA). For the sample selection, first, we identified all salaried workers who had had an SA due to a malignant neoplasm (ICD-10, C00-C97) between 2012 and 2015 from anonymized ICAM records (*N* = 645), and they were linked to the information of the WORKss cohort (156,000 salaried workers affiliated with the general scheme of social security in Catalonia), resulting in 516 workers with information in both databases. Second, for each worker with SA due to cancer, we individually matched two workers by age (within a 5-year range), sex (men and women), and time at risk (cohort entry in the same week as the SA due to cancer ended). First, a total of 516 salaried workers with an SA due to a medical diagnosis other than cancer (ending the same week as the SA due to cancer) were randomly selected from a group of 47,663 workers in the WORKss cohort among those who met the individual matching criteria. Second, 516 salaried workers without an SA were randomly selected from the WORKss cohort among those who met the individual matching criteria after excluding workers who had an SA for any medical diagnosis at the beginning of follow-up (*N* = 139,744) (Supplementary Tables 1 and 2). The selection of two comparison groups with and without SA was made to assess a potential gradient of the possible effect of cancer on working life with the general working population and with workers with other health problems recognised by SA.

The average age of the three comparison groups in 2012 was 49.8 years for men (standard deviation: 9.96) and 47.0 years for women (standard deviation: 9.44). Among the comparison group of workers with an SA for reasons other than cancer, the most frequent cause was diseases of the musculoskeletal system and connective tissue in both men and women (Supplementary Table 2).

Each worker’s working life was characterised weekly from the time they entered the cohort between 2012 and 2015 until December 31, 2018, according to the possible labour states after an SA due to cancer. The study period ranged from 3 to 7 years, and the time out of the cohort was censored. The possible states were temporary employment, permanent employment, unemployment with benefits, inactivity (considered as not having contact with social security longer than 15 days), PD (including total, absolute and severe degrees), early retirement, ordinary retirement, and death. Two retirement states were defined according to the Spanish social security modalities: ordinary and early retirement. Briefly, in Spain, to retire with a full pension, a worker must be the legal retirement age (65 years old) and have contributed 37 years to social security [19]. To retire early, a worker must be no more than two years younger than the legal retirement age and have contributed at least 35 years. Other scenarios also entitle individuals to retire early, such as having a disability or working in a high-mortality occupation, such as mining, among others [20]. If a worker was in two different states in the same week, that worker was assigned the state where he or she spent the longest time.

Potential confounders included in our analysis were occupational category (nonmanual skilled, nonmanual nonskilled, manual skilled, or manual nonskilled); working time categorised as a percentage of weekly hours (full-time [> 87.5%], part-time [50%-87.5%], or short and marginal part-time [≤ 37.5%-49%]); monthly average income in tertiles based on the income of the study population in 2012 (high [> €2370], medium [€1450–2370], or low [≤ €1450]); company size (small/medium [up to 100 employees] and large [> 100 employees]); company ownership (private and public); and the company’s economic activity (primary sector [agriculture, hunting, forestry, fishing, mining, and quarrying]; manufacturing [including construction and energy], and services). We also considered the previous 5-year employment ratio expressed as a percentage of employed days to the total potential working days, including working or unemployed or unaffiliated days, to assess attachment to the labour market before SA due to cancer. Workers who changed categories over time were assigned the category in which they spent most of the follow-up period.

Patients were not involved in any stage of this study, and confidentiality was maintained in both databases. The authors received data that were previously anonymised.

### Statistical analysis

The sample was described according to the abovementioned response variables, explanatory variables and covariates, and the chi-square test was employed to assess the significance between comparison groups (Supplementary Table 1). Sequence analysis was performed to reconstruct individual working lives by generating an ordered list of weekly labour states after an SA due to cancer. Optimal matching and cluster analyses were applied to identify groups of workers who shared similar working life trajectories. We called the resulting trajectories future labour market participation patterns (LMPPs). Average silhouette width (ASW) was used to select the optimal number of clusters (values higher than 0.51 are recommended; Supplementary Table 3) [21, 22].

To measure the association between having an SA due to cancer and future LMPPs versus the comparison groups, we applied multinomial logistic regression with its relative risk ratio (RRR) and 95% confidence interval (95% CI) using the stable employment LMPP as a reference.

All analyses were stratified by sex. Stata v.13 software was used for multinomial regression models, and R v.4.1.0 was used for sequence analysis and optimal matching analysis.

## Results

**Fig. 2:**
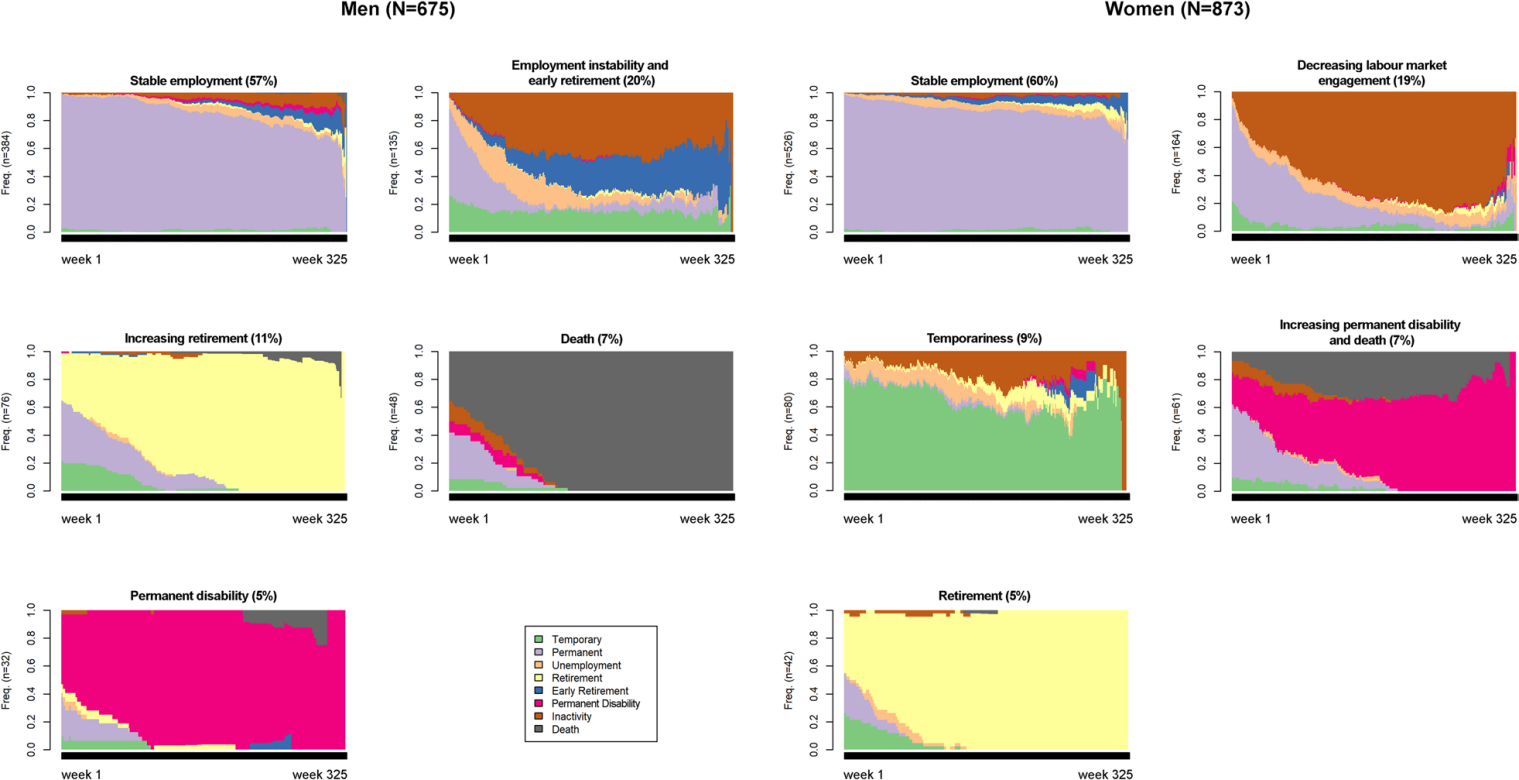
Future labour market participation patterns (LMPPs) in a sample of salaried men (top) and women (bottom) in Catalonia (2012–2018)

We found five LMPPs for both sexes, which were named as a description of the labour trajectories found for each sex. Among women, shared LMPPs were stable employment (60.3%), decreasing labour market engagement (18.7%), temporariness (9.1%), increasing PD and death (7.0%), and retirement (4.8%). Men’s future LMPPs were summarised as stable employment (56.9%), employment instability and early retirement (20.0%), increasing retirement (11.4%), death (7.1%), and PD (4.7%) (Fig. 2, Table 1).

**Table 1:** Future labour market participation patterns (LMPPs) among salaried workers living in Catalonia (2012–2018)

	**Employment trajectories**											
	**Women (*N* = 873)**						**Men (*N* = 675)**					
	**Stable employment (60.3%)**	**Decreasing labour market engagement (18.7%)**	**Temporariness (9.1%)**	**Increasing PD and death (7%)**	**Retirement (4.8%)**	***p* value**	**Stable employment (56.9%)**	**Employment instability and early retirement (20%)**	**Increasing retirement (11.4%)**	**Death (7.1%)**	**PD (4.7%)**	***p* value**
	**N (%)**	**N (%)**	**N (%)**	**N (%)**	**N (%)**		**N (%)**	**N (%)**	**N (%)**	**N (%)**	**N (%)**	
**Comparison groups**												
SA-cancer	170 (32.3)	42 (25.6)	27 (33.8)	38 (62.3)	14 (33.3)		118 (30.7)	32 (23.7)	25 (32.9)	42 (87.5)	8 (25.0)	
SA-other diagnoses	169 (32.1)	65 (39.6)	29 (36.3)	15 (24.6)	13 (31.0)	< 0.0001***	120 (31.3)	57 (42.2)	27 (35.5)	5 (10.4)	16 (50.0)	< 0.0001***
No-SA for any diagnoses	187 (35.6)	57 (34.8)	24 (30.0)	8 (13.1)	15 (35.7)		146 (38.0)	46 (34.1)	24 (31.6)	1 (2.1)	8 (25.0)	
**Follow-up period**												
**Age in 2012 (years)**												
≤ 25	3 (0.6)	4 (2.44)	2 (2.5)	*	*	< 0.0001***	3 (0.8)	3 (2.2)	*	*	*	< 0.0001***
26–35	63 (12.0)	24 (14.6)	9 (11.3)	6 (8.8)	*		49 (12.8)	18 (13.3)	*	3 (6.3)	2 (6.3)	
36–45	186 (35.4)	55 (33.5)	23 (28.8)	18 (29.5)	*		93 (24.2)	18 (13.3)	*	8 (16.7)	1 (3.1)	
46–55	200 (38.0)	57 (34.8)	25 (31.3)	24 (39.4)	*		159 (41.4)	46 (34.1)	*	13 (27.1)	10 (31.3)	
> 55	74 (14.1)	24 (14.6)	21 (26.3)	13 (21.3)	42 (100)		20 (20.8)	50 (37.0)	76 (100)	24 (50.0)	19 (59.4)	
**Average monthly income (tertiles)**												
High (> €2370.0)	167 (31.8)	27 (16.5)	14 (17.5)	12 (19.7)	3 (7.1)	< 0.0001***	196 (51.0)	46 (34.1)	24 (31.6)	15 (31.3)	5 (15.6)	< 0.0001***
Medium (€1451.0–2370.0)	197 (37.5)	42 (25.6)	34 (42.5)	13 (19.7)	6 (14.3)		137 (35.7)	44 (32.6)	16 (21.1)	10 (20.8)	11 (34.4)	
Low (≤ €1450.0)	162 (30.8)	91 (55.5)	32 (40.0)	31 (50.8)	33 (78.6)		51 (13.3)	43 (31.9)	35 (46.1)	19 (39.6)	13 (40.6)	
**Occupational category**												
Nonmanual skilled	136 (25.9)	31 (18.9)	22 (27.5)	10 (16.4)	16 (38.1)	0.003**	89 (23.2)	29 (21.5)	26 (34.2)	9 (18.75)	3 (9.4)	0.039*
Nonmanual nonskilled	246 (46.8)	59 (36.0)	35 (43.8)	27 (44.3)	12 (28.6)		123 (32.0)	44 (32.6)	15 (19.7)	18 (37.5)	12 (37.5)	
Manual skilled	76 (14.5)	28 (17.1)	9 (11.3)	14 (23.0)	3 (7.1)		139 (36.2)	45 (33.3)	26 (34.2)	11 (22.9)	11 (34.4)	
Manual nonskilled	51 (9.7)	29 (17.7)	8 (10.0)	8 (13.1)	10 (23.8)		22 (5.7)	12 (8.9)	8 (10.5)	2 (4.2)	6 (18.8)	
**Company economic activity**												
Manufacturing, energy, construction	84 (16.0)	16 (9.8)	6 (7.5)	6 (9.8)	1 (1.4)	0.016*	123 (32.0)	38 (28.2)	24 (31.6)	11 (22.9)	12 (37.5)	0.717
Services	432 (82.1)	140 (85.4)	73 (91.3)	51 (83.6)	41 (97.6)		253 (65.9)	91 (67.4)	47 (61.8)	31 (64.6)	18 (56.3)	
**Working time** (% weekly hours)												
Full-time (> 87.5%)	423 (80.4)	111 (67.7)	52 (65.0)	40 (66.7)	11 (26.2)	< 0.0001***	363 (94.5)	117 (86.7)	31 (40.8)	33 (68.8)	25 (78.1)	< 0.0001***
Part-time (50%-87.5%)	89 (16.9)	33 (20.1)	11 (13.8)	15 (25.0)	4 (9.5)		14 (3.7)	8 (5.9)	5 (6.6)	4 (8.3)	5 (15.6)	
Short and marginal part-time (≤ 37.5%-49%)	14 (2.7)	19 (11.6)	17 (21.3)	5 (8.3)	27 (64.3)		7 (1.8)	10 (7.4)	39 (51.3)	5 (10.4)	2 (6.3)	
**Company size**												
Small-medium (≤ 100 workers)	269 (51.1)	112 (68.7)	36 (45.0)	29 (48.3)	25 (59.5)	0.001**	229 (59.6)	92 (68.2)	49 (64.5)	23 (47.9)	24 (75.0)	0.156
Large (> 100 workers)	257 (48.9)	51 (31.3)	44 (55.0)	31 (51.7)	17 (40.5)		155 (40.4)	43 (31.9)	26 (34.2)	19 (39.6)	8 (25.0)	
**Company ownership**												
Private	373 (70.9)	108 (65.9)	37 (46.3)	42 (68.9)	33 (78.6)	< 0.0001***	303 (78.9)	98 (72.6)	55 (72.4)	32 (66.7)	27 (84.4)	0.956
Public	102 (19.4)	22 (13.4)	32 (40.0)	11 (18.0)	7 (16.7)		59 (15.4)	19 (14.1)	13 (17.1)	6 (12.5)	4 (12.5)	
**5 years prior to follow-up**												
	**Mean (SD)**	**Mean (SD)**	**Mean (SD)**	**Mean (SD)**	**Mean (SD)**		**Mean (SD)**	**Mean (SD)**	**Mean (SD)**	**Mean (SD)**	**Mean (SD)**	
**Employment time (ratio)**	95.1 (12.9)	87.0 (22.7)	86.3 (23.6)	89.6 (18.6)	97.4 (9.3)	< 0.0001***	95.0 (13.6)	86.1 (23.4)	97.3 (10.1)	84.3 (29.1)	88.3 (21.1)	< 0.0001***
**Total**	526	164	80	61	42		384	135	76	48	32	

*SA* Sickness absence; The follow-up period ranged from 3 to 7 years, from cohort entry until the end of 2018; previous 5 years, calculated from each individual’s entry, *SD* Standard deviation

*p < 0.05; **p < 0.01; ***p < 0.001

For both sexes, the most frequent LMPP was stable employment. Among workers with an SA due to cancer, 32.3% of women and 30.7% of men showed this pattern (Table 1). In this LMPP, 80% of workers were employed on a permanent contract throughout the follow-up period (Fig. 2), and monthly income tended to be high (31.8% of women and 51.0% of men). Women also showed the lowest proportion of manual nonskilled occupations (9.7%) (Table 1).

Taking this stable employment LMPP as the reference category for both sexes and compared with workers with an SA due to cancer, we examined the probability of belonging to each LMPP among workers with an SA due to other causes and workers without an SA (Tables 2 and 3).

**Table 2:** Association among future labour market participation patterns (LMPPs), company characteristics, and employment conditions among female salaried workers with an SA due to cancer (reference) and comparison groups

	**Women**											
	**Decreasing labour market engagement vs. stable employment**			**Temporariness vs. stable employment**			**Retirement vs. stable employment**			**Increasing PD and death vs. stable employment**		
	**RRR**	**95% CI**	***p* value**	**RRR**	**95% CI**	***p* value**	**RRR**	**95% CI**	***p* value**	**RRR**	**95% CI**	***p* value**
**Crude**												
SA-cancer	1			1			1			1		
SA-other diagnoses	1.56	(1.00‒2.42)	0.050	1.08	(0.61‒1.90)	0.789	0.93	(0.43‒2.05)	0.865	0.40	(0.21‒0.75)	0.004**
No-SA for any diagnoses	1.23	(0.78‒1.93)	0.360	0.81	(0.45‒1.45)	0.477	0.97	(0.46‒2.08)	0.946	0.19	(0.09‒0.42)	< 0.0001***
**Individually adjusted by**												
**Income (tertiles)**												
SA-other diagnoses	1.55	(0.98‒2.45)	0.059	1.06	(0.60‒1.87)	0.850	0.92	(0.41‒2.05)	0.831	0.42	(0.22‒0.83)	0.012*
No-SA for any diagnoses	1.12	(0.70‒1.78)	0.630	0.79	(0.43‒1.42)	0.425	0.84	(0.38‒1.82)	0.650	0.20	(0.09‒0.45)	< 0.0001***
**Occupational category**												
SA-other diagnoses	1.47	(0.92‒2.36)	0.111	1.08	(0.60‒1.94)	0.808	1.01	(0.45‒2.25)	0.983	0.32	(0.16‒0.62)	0.001**
No-SA for any diagnoses	1.21	(0.75‒1.95)	0.435	0.77	(0.42‒1.43)	0.414	0.96	(0.44‒2.11)	0.926	0.18	(0.08‒0.40)	< 0.0001***
**Company economic activity**												
SA-other diagnoses	1.67	(1.06‒2.64)	0.027*	1.11	(0.62‒1.96)	0.725	1.03	(0.47‒2.26)	0.944	0.41	(0.21‒0.80)	0.009**
No-SA for any diagnoses	1.37	(0.86‒2.17)	0.185	0.87	(0.48‒1.57)	0.643	1.07	(0.50‒2.29)	0.862	0.22	(0.10‒0.49)	< 0.0001***
**Working time (% weekly hours)**												
SA-other diagnoses	1.58	(1.01‒2.47)	0.047*	1.03	(0.58‒1.83)	0.923	0.80	(0.33‒1.92)	0.610	0.41	(0.22‒0.77)	0.006**
No-SA for any diagnoses	1.26	(0.80‒1.99)	0.321	0.78	(0.43‒1.42)	0.410	0.86	(0.37‒2.03)	0.732	0.20	(0.09‒0.44)	< 0.0001***
**Company size**												
SA-other diagnoses	1.68	(1.07‒2.64)	0.023*	1.06	(0.60‒1.87)	0.834	0.96	(0.44‒2.10)	0.914	0.40	(0.21‒0.77)	0.005*
No-SA for any diagnoses	1.24	(0.79‒1.96)	0.352	0.81	(0.45‒1.46)	0.490	0.97	(0.45‒2.06)	0.929	0.20	(0.09‒0.44)	< 0.0001***
**Company ownership**												
SA-other diagnoses	1.65	(1.01‒2.71)	0.045*	1.14	(0.62‒2.12)	0.675	1.00	(0.45‒2.23)	0.999	0.41	(0.21‒0.81)	0.010*
No-SA for any diagnoses	1.35	(0.82‒2.24)	0.242	0.93	(0.49‒1.77)	0.826	1.04	(0.47‒2.27)	0.931	0.24	(0.11‒0.54)	0.001**
**Previous 5-year employment time (ratio)**												
SA-other diagnoses	1.62	(1.03‒2.54)	0.036*	1.13	(0.64‒2.00)	0.683	0.92	(0.42‒2.02)	0.833	0.41	(0.22‒0.77)	0.006*
No-SA for any diagnoses	1.26	(0.80‒2.00)	0.320	0.81	(0.44‒1.47)	0.488	0.97	(0.45‒2.07)	0.937	0.20	(0.09‒0.43)	< 0.0001***
**Adjustment by all variables**												
SA-other diagnoses	1.72	(1.02‒2.90)	0.043*	1.09	(0.56‒2.13)	0.801	0.72	(0.26‒1.95)	0.515	0.39	(0.19‒0.83)	0.014*
No-SA for any diagnoses	1.23	(0.72‒2.11)	0.442	0.90	(0.45‒1.80)	0.762	0.60	(0.22‒1.65)	0.324	0.24	(0.10‒0.57)	0.001**

*SA* Sickness absence, *PD* Permanent disability, *RRR* Relative risk ratio, *95% CI* 95% confidence interval

^*^p < 0.05; **p < 0.01; ***p < 0.001

**Table 3:** Association among future labour market participation patterns (LMPPs), company characteristics, and employment conditions among male salaried workers with an SA due to cancer (reference) and comparison groups

	**Men**											
	**Employment instability and early retirement vs. stable employment**			**Increasing retirement vs. stable employment**			**PD vs. stable employment**			**Death vs. stable employment**		
	**RRR**	**95% CI**	***p* value**	**RRR**	**95% CI**	***p* value**	**RRR**	**95% CI**	***p* value**	**RRR**	**95% CI**	***p* value**
**Crude**												
SA-cancer	1			1			1			1		
SA-other diagnoses	1.75	(1.06‒2.89)	0.029*	1.06	(0.58‒1.94)	0.844	1.97	(0.81‒4.77)	0.135	0.12	(0.04‒0.31)	< 0.0001^***^
No-SA for any diagnoses	1.16	(0.70‒1.94)	0.566	0.78	(0.42‒1.43)	0.415	0.81	(0.29‒2.22)	0.679	0.02	(0.00‒0.14)	< 0.0001^***^
**Individually adjusted by**												
**Income (tertiles)**												
SA-other diagnoses	1.68	(1.00‒2.79)	0.048*	1.06	(0.57‒1.98)	0.857	2.22	(0.82‒6.02)	0.116	0.12	(0.05‒0.33)	< 0.0001^***^
No-SA for any diagnoses	1.03	(0.61‒1.74)	0.920	0.73	(0.38‒1.38)	0.334	0.92	(0.31‒2.77)	0.881	0.02	(0.00‒0.14)	< 0.0001^***^
**Occupational category**												
SA-other diagnoses	1.63	(0.98‒2.73)	0.062	1.17	(0.63‒2.18)	0.611	1.77	(0.72‒4.36)	0.212	0.14	(0.05‒0.38)	< 0.0001^***^
No-SA for any diagnoses	1.18	(0.70‒1.99)	0.526	0.87	(0.47‒1.63)	0.667	0.80	(0.29‒2.21)	0.666	0.02	(0.00‒0.18)	< 0.0001^***^
**Company economic activity**												
SA-other diagnoses	1.92	(1.14‒3.22)	0.013*	1.07	(0.58‒1.98)	0.836	2.19	(0.86‒5.57)	0.100	0.14	(0.05‒0.37)	< 0.0001^***^
No-SA for any diagnoses	1.21	(0.71‒2.05)	0.481	0.72	(0.38‒1.37)	0.320	0.82	(0.28‒2.40)	0.713	0.02	(0.00‒0.17)	< 0.0001^***^
**Working time (% weekly hours)**												
SA-other diagnoses	1.78	(1.08‒2.95)	0.025*	1.33	(0.65‒2.72)	0.440	1.99	(0.81‒4.86)	0.132	0.14	(0.05‒0.37)	< 0.0001^***^
No-SA for any diagnoses	1.13	(0.68‒1.89)	0.641	0.66	(0.31‒1.40)	0.278	0.78	(0.28‒2.16)	0.635	0.02	(0.00‒0.16)	< 0.0001^***^
**Company size**												
SA-other diagnoses	1.76	(0.06‒2.91)	0.028*	1.11	(0.61‒2.03)	0.737	1.98	(0.82‒4.82)	0.131	0.14	(0.05‒0.36)	< 0.0001^***^
No-SA for any diagnoses	1.13	(0.67‒1.88)	0.650	0.79	(0.43‒1.47)	0.457	0.76	(0.28‒2.10)	0.602	0.02	(0.00‒0.17)	< 0.0001^***^
**Company ownership**												
SA-other diagnoses	1.53	(0.90‒2.59)	0.115	1.03	(0.55‒1.94)	0.922	2.19	(0.87‒5.54)	0.098	0.15	(0.06‒0.40)	< 0.0001^***^
No-SA for any diagnoses	1.14	(0.66‒1.94)	0.643	0.79	(0.41‒1.50)	0.470	0.95	(0.33‒2.72)	0.930	0.03	(0.00‒0.19)	< 0.0001^***^
**Previous 5-year employment time (ratio)**												
SA-other diagnoses	1.86	(1.11‒3.11)	0.018*	1.05	(0.58‒1.91)	0.876	2.06	(0.84‒5.01)	0.113	0.12	(0.05‒0.33)	< 0.0001^***^
No-SA for any diagnoses	1.24	(0.73‒2.09)	0.431	0.79	(0.43‒1.45)	0.448	0.85	(0.31‒2.34)	0.750	0.02	(0.00‒0.15)	< 0.0001^***^
**Adjustment by all variables**												
SA-other diagnoses	1.76	(1.00‒3.11)	0.052	1.38	(0.61‒3.14)	0.443	2.24	(0.79‒6.34)	0.128	0.17	(0.06‒0.46)	0.001^**^
No-SA for any diagnoses	1.09	(0.61‒1.96)	0.761	0.64	(0.27‒1.52)	0.314	0.86	(0.27‒2.75)	0.793	0.02	(0.00‒0.16)	< 0.0001^***^

*SA* Sickness absence, *PD* Permanent disability, *RRR* Relative risk ratio, *95% CI* 95% confidence interval

^*^p < 0.05; ^**^p < 0.01; ^***^p < 0.001

Table 1 shows how the second most frequent LMPP was employment instability and early retirement among men and decreasing labour market engagement among women. These LMPPs represented a group of workers mostly employed on permanent contracts (60.0%), but very soon they switched to inactivity among women, whereas among men, the switch was towards unemployment benefits and inactivity or to early retirement (Fig. 2). Table 2 shows that, taking workers with an SA due to cancer as a reference group, women with an SA due to other causes had a higher probability of being in a decreasing labour market engagement LMPP rather than in a stable employment LMPP (aRRR 1.72, 95% CI: 1.02‒2.90). Among men (Table 3), a similar pattern was found for the employment instability and early retirement LMPPs, with the same differences between workers with SA due to other causes and those with SA due to cancer (aRRR 1.76, 95% CI: 1.00‒3.11).

Both men and women showed LMPPs characterised by a group of workers who died during follow-up: the death LMPP for men and increasing permanent disability and death LMPPs for women. For men, this pattern depicting workers who died during follow-up consisted mainly of workers with an SA due to cancer. However, the pattern showing workers on PD was more frequent among men with an SA due to other causes (50.0%) and those aged over 55 years (55.6%) (Table 1). For women, the LMPP of increasing PD and death was less probable in comparison groups (no SA, aRRR 0.24, 95% CI: 0.10‒0.57; SA due to other causes, aRRR 0.39, 95% CI: 0.19‒0.83) (Table 2). Among men, as well as among women, both comparison groups showed a lower probability of death (no SA, aRRR 0.02, 95% CI: 0.00‒0.16; SA due to other causes, aRRR 0.17, 95% CI: 0.06‒0.46) (Table 3).

Among women, unlike among men, an LMPP of temporariness represented 9% of the sample (Fig. 2). This LMPP showed the lowest percentage of employed people in the 5 years before entering the cohort (86.3%) (Table 1). Women without an SA showed trends towards a lower likelihood of being in a temporariness pattern (aRRR 0.90, 95% CI: 0.45‒1.80) (Table 2).

We found LMPPs of increasing retirement and retirement for men and women, respectively (Table 1, Fig. 2). For men, this LMPP was the third most prevalent (11.4%), with almost 100% of men being retired by the end of follow-up. Women showed a similar LMPP, but in contrast, it was the least frequent pattern (Table 1). Both men and women in these patterns were over 55 years of age and worked in a private company (Table 1). When we assessed the probability of having an LMPP of exit from the labour market due to retirement, workers without an SA showed trends of a lower likelihood of being in this pattern, especially when adjusted by all employment and working conditions, even if they were not statistically significant (women, aRRR 0.60, 95% CI: 0.22‒1.65; men, aRRR 0.64, 95% CI: 0.27‒1.52) compared to those with an SA due to cancer (Tables 2 and 3).

## Discussion

The results of this study support our hypothesis that workers with an SA due to cancer were more likely to retire, receive PD benefits or die than their counterparts. However, patterns depicting these outcomes represented a small proportion of the sample, and the results should be interpreted with caution. Our findings were consistent after adjustment for several employment and working conditions.

Our results on ordinary retirement among workers with an SA due to cancer, even if they were not statistically significant, are in line with those of previous studies. Patterns depicting ordinary retirement were more likely among cancer survivors. These results showed small estimates and were not statistically significant, especially for women. For men, this trend was found only compared to those without SA. However, although they should be interpreted with scepticism, these results are important in terms of the population they represent. As previous studies have shown, workers very close to retirement age who are diagnosed with cancer may choose to retire [23]. Cancer treatments are still, in general, highly aggressive and leave some survivors with long-term health problems that may make them unable to carry out their prior job duties. For example, a systematic review found that long-term survivors were less likely to be working than people without cancer [24]. Some workers experience less severe side effects from treatment but require longer SAs for recovery and readaptation to be able to work. In this regard, the social security system plays a major role by setting a maximum amount of SA time that does not always suit cancer survivors’ needs. These results question the effectiveness of the Spanish social security system in maintaining workers in the labour market during their working life and, when they are ill, in guaranteeing their income through benefits. In this case, after cancer treatment, some workers could continue working but need more flexible SA schemes to recover from the range of effects produced by cancer. Another explanation could be that workers who have had a serious health problem may find it more difficult to maintain the pace of a normal full-time job in the long term. However, they might also encounter discrimination, including hiring discrimination, harassment, job reassignment, job loss, and limited career advancement, due to their health problems [25]. These results are also consistent with the well-known healthy worker effect [26].

When we compared the probability of having a stable future working life to other patterns, we found unexpected results among workers with an SA due to cancer. Men, in general, were less likely to be in unstable or early retirement or PD trajectories than those with SA due to other causes. Although not statistically significant and with lower estimates, the same result was found for instability and early retirement trajectories compared to workers without an SA. Among women, we observed similar statistically nonsignificant tendencies of decreasing labour market engagement in the two comparison groups. Among those with an SA due to other causes, these estimates became significant after adjusting for employment-related conditions. For both sexes, individual adjustment by the previous five-year attachment to the labour market increased the estimations, as did the economic activity of the company and the working time. In light of these results, we hypothesised that future instability could lie in the fact that SA due to other causes was highly represented by diseases of the musculoskeletal system and connective tissue, and these types of diagnoses are more prevalent among manual workers [27, 28], who normally have more precarious jobs characterised by temporality and insecurity in Catalonia [29] and in the whole of Spain [30]. Few studies have assessed RTW and long-term working trajectories regarding PD or early retirement after all-cause SA among workers comparing them with those with an SA due to cancer. A study carried out in Norway with a 10-year follow-up that examined long-term SA due to all diagnoses found that 32% of workers with SA due to any diagnoses had low attachment labour market trajectories, consisting of part-time work, recurrence of SA, unemployment or PD [31]. Having a comparison group with workers who have other health conditions, also recognised by an SA, allowed us to analyse future differences in working life between overcoming cancer and other health problems, and how after ending SA due to the adverse effects of cancer, the ability to work of survivors may still be affected, even more than other health problems. Moreover, this allowed us to control for the potential effect of SA itself, as SA is a determinant of low labour engagement and future SA [32].

This study identified differences in employment patterns by sex. Only for women did we find an LMPP of temporary employment. This LMPP was more probable among workers with an SA due to cancer than among workers with no SA. In Spain, temporary and part-time employment is more prevalent among women due to enormous gender inequities in the labour market [33]. A similar study carried out by Pedersen et al. in Scandinavian countries using the same methodology but examining employment trajectories after all-cause SA found no differences by sex [34], whereas another study by Madsen on labour market trajectories after RTW from a long-term SA found that women were more prone to be in less stable trajectories with part-time work and SA recurrence than men [31]. It is likely that this sex-based temporariness was exacerbated by cancer. However, the differences were not statistically significant, so future studies should look at future precarity among women who are cancer survivors.

We also found differences by sex in retirement. For men, the difference in the likelihood of retirement among workers with an SA due to cancer compared with those without an SA was larger than that for women. We found a percentage of male workers who started to retire early, and this percentage increased steadily during the follow-up period. Additionally, the retirement pattern was twice as prevalent among men as among women. This might be due to differences in ease of access to retirement for several reasons. First, as in all countries, a minimum period of pension contributions is a prior condition for retirement or early retirement. Due to reproductive work and motherhood, women often contribute fewer years to the social security system, and for early retirement, workers must have contributed for at least 35 years [35, 36]. Second, men are generally in better paid jobs, and since the amount of the early retirement pension depends on the workers’ previous regulatory basis, men have better early retirement pensions, making retirement more appealing [33]. In addition, some companies have early retirement policies if the worker voluntarily wants to retire and does not meet social security requirements, and these policies are more accessible to men than to women [37], probably because they exist in male-dominated sectors. Last, in our study, the sample of men was older than that of women, and throughout Spain, to retire before retirement age, the worker must be a maximum of 2 years below this age to obtain full pension benefits.

The main limitation of the present study was the lack of information on cancer other than that diagnosed for SA from 2012–2015. Hence, we were unable to take into consideration the effect of clinical features (i.e., type of treatment, stage of cancer, effects of health status before 2012). In addition, we managed time-varying covariates by assigning workers to the category in which they spent most of their time during the follow-up. Additionally, self-employed individuals were not included in this study because they did not have access to SA benefits during the study period. Furthermore, the methodology applied to employment trajectories involved an algorithmic data-driven approach that classified individuals according to similar characteristics. Consequently, some resulting groups had a small number of observations, which should be interpreted cautiously.

The primary strength of this study, in terms of occupational health relevance, as well as from a clinical perspective, is the use of administrative data with information on social security benefits and an extensive follow-up with information on labour market states, which is needed to obtain a sufficient overview of the RTW process. The size of the database allowed us to match our workers with SA due to cancer to two comparison groups by age, sex, and follow-up time. This matching allowed us to compare the working life of cancer survivors with that of the general working population with or without SAs, enabling us to account not only for the disease but also for the effect of SA. The diagnoses that resulted in SAs were medically certified by primary care doctors rather than self-reported, enhancing the validity of our results [38].

## Conclusions

Most workers with an SA due to cancer have a future working trajectory in employment. However, our results showed that they are more likely to die during their working life than their counterparts of the same age and sex. We also found upward trends of retiring or receiving PD benefits compared to the general working population from a life course perspective, further studies with larger sample sizes are necessary to assess these results. This study represents a step towards a deeper understanding of the consequences of cancer on working life, as it captured all possible labour outcomes and their chronological onset in workers’ lives. However, more studies are needed to address questions that remain unanswered in light of our results, such as whether there are any differences between cancer diagnoses, stages, or treatments regarding future working trajectories.

Nevertheless, for the time being, action should be taken to regulate programmes, many of which are common to all diagnoses, in the workplace that consider the needs of workers who have survived cancer, so that these workers may still work when possible and desired.

### Supplementary Information

**Additional file 1: Supplementary Table 1.** Employment-related characteristics among a sample of salaried workers with a SA due to cancer, SA due to other diagnoses, or no SA at all in Catalonia during the follow-up period (2012 and 2018), and previous employment 5 years prior to cohort entrance. **Supplementary Table 2.** Cancer location in the group of workers with SA due to cancer and diagnosis underlying SAs in the comparison group with other causes by sex in Catalonia (2012-2015). **Supplementary Table 3.** Selection of cluster solution and cluster quality measure Average Silhouette Width (ASW) for future working life of a sample of salaried workers in Catalonia (2012-2018) with 8 possible states.

## Data Availability

The data that support the findings of this study are available from the General Directorate of Social Security (DGOSS by its acronym in Spanish) and the Catalan Institute for Medical and Health Evaluations (ICAM by its acronym in Catalan), but restrictions apply to the availability of these data, which were used under licence for the current study and so are not publicly available. Data should be requested from the two corresponding institutions at https://www.seg-social.es/wps/portal/wss/internet/EstadisticasPresupuestosEstudios/Estadisticas/EST211 for working life data and bustia.icam@gencat.cat for data on SA.

## Abbreviations

SA: Sickness absence
LMPP: Labour market participation pattern
RRR: Relative risk ratio
aRRR: Adjusted relative risk ratio
95% CI: 95% Confidence interval
EU: European Union
RTW: Return to work
PD: Permanent disability
ICD-10: 10Th Revision of the International Classification of Diseases
ASW: Average silhouette width
