# Supplementary material for: Labour market participation after sickness absence due to cancer: a dynamic cohort study in Catalonia (Spain)
Source: BMC Public Health. 2023 Dec 11;23:2477. doi: 10.1186/s12889-023-17321-z (PMC10714569; doi:10.1186/s12889-023-17321-z)
Supplement: Supplementary file 1 — Additional file 1: Supplementary Table 1. Employment-related characteristics among a sample of salaried workers with a SA due to cancer, SA due to other diagnoses, or no SA at all in Catalonia during the follow-up period (2012 and 2018), and previous employment 5 years prior to cohort entrance. Supplementary Table 2. Cancer location in the group of workers with SA due to cancer and diagnosis underlying SAs in the comparison group with other causes by sex in Catalonia (2012-2015). Supplementary Table 3. Selection of cluster solution and cluster quality measure Average Silhouette Width (ASW) for future working life of a sample of salaried workers in Catalonia (2012-2018) with 8 possible states. [file 12889_2023_17321_MOESM1_ESM.docx]

| SUPLEMENTARY MATERIAL:  *Supplementary table 1: Employment-related characteristics among a sample of salaried workers with a SA due to cancer, SA due to other diagnoses, or no SA at all in Catalonia during the follow-up period (2012 and 2018), and previous employment 5 years prior to cohort entrance.* | | | | | | | | | | | |  |  |
| --- | --- | --- | --- | --- | --- | --- | --- | --- | --- | --- | --- | --- | --- |
|  |  |  |  |  |  |  |  |  |  |  |  |  | |
|  |  |  |  |  |  |  |  |  |  | |  | |  |
|  | **Men (N=675)** | | |  | **Women (N=873)** | | | | |  | |  | |
|  | **SA-cancer (N=225)** | **SA-other diagnoses (N=225)** | **No SA any diagnoses (N=225)** |  | **SA-cancer (N=291)** | **SA-other diagnoses (N=291)** | **No SA any diagnoses (N=291)** |  |  | |  | |  |
| **Follow-up period** |  |  |  |  |  |  |  |  |  | |  | |  |
| **Total accumulated days of employment** | 262,869 | 286,245 | 328,939 |  | 393,825 | 425,556 | 434,249 |  |  | |  | |  |
|  | **N (%)** | **N (%)** | **N (%)** | **p value** | **N (%)** | **N (%)** | **N (%)** | **p value** |  | |  | |  |
| **Contract type** |  |  |  | |  |  |  | | |  |  | |  |
| Permanent | 178 (79.1) | 188 (83.6) | 194 (86.2) | 0.003** | 243 (83.5) | 233 (80.1) | 243 (83.5) | 0.206 |  | |  | |  |
| Temporary | 40 (17.8) | 37 (16.4) | 31 (13.8) |  | 46 (15.8) | 58 (19.9) | 48 (16.5) |  |  | |  | |  |
| **Working time** (% weekly hours) |  |  |  |  |  |  |  |  |  | |  | |  |
| Full-time (>87.5%) | 184 (81.8) | 195 (86.7) | 190 (84.4) | 0.037* | 206 (70.8) | 213 (73.2) | 218 (74.9) | 0.188 |  | |  | |  |
| Part-time (50%-87.5%) | 12 (5.3) | 13 (5.8) | 11 (4.9) |  | 59 (20.3) | 48 (16.5) | 45 (15.5) |  |  | |  | |  |
| Short and marginal part-time (≤37.5%-49%) | 22 (9.8) | 17 (7.6) | 24 (10.7) |  | 24 (8.2) | 30 (10.3) | 28 (9.6) |  |  | |  | |  |
| **Monthly average income (tertiles)** |  |  |  |  |  |  |  |  |  | |  | |  |
| High (>2370.0 €) | 105 (48.2) | 90 (40.2) | 91 (40.8) | 0.364 | 79 (27.7) | 70 (24.2) | 74 (25.6) | 0.360 |  | |  | |  |
| Medium (1451.0 - 2370.0 €) | 61 (28.0) | 79 (35.3) | 78 (35.0) |  | 97 (34.0) | 107 (37.0) | 87 (30.1) |  |  | |  | |  |
| Low (≤1450.0 €) | 52 (23.9) | 55 (24.6) | 54 (24.2) |  | 109 (38.3) | 112 (38.8) | 128 (44.3) |  |  | |  | |  |
| **Occupational category** |  |  |  |  |  |  |  |  |  | |  | |  |
| Non-manual skilled | 67 (29.8) | 38 (16.9) | 51 (22.7) | <0.0001*** | 94 (32.3) | 53 (18.2) | 68 (23.4) | <0.0001*** |  | |  | |  |
| Non-manual non-skilled | 74 (32.9) | 69 (30.7) | 69 (30.7) |  | 129 (44.3) | 129 (44.3) | 121 (41.6) |  |  | |  | |  |
| Manual skilled | 59 (26.2) | 91 (40.4) | 82 (36.4) |  | 29 (10.0) | 57 (19.6) | 44 (15.1) |  |  | |  | |  |
| Manual non-skilled | 14 (6.2) | 21 (9.3) | 15 (6.7) |  | 28 (9.6) | 40 (13.8) | 38 (13.1) |  |  | |  | |  |
| **Economic activity** |  |  |  |  |  |  |  |  |  | |  | |  |
| Agriculture, hunting, forestry, fishing, mining, and quarrying | 1 (0.4) | * | 4 (1.8) | 0.002** | 1 (0.3) | * | 2 (0.7) | 0.130 |  | |  | |  |
| Manufacturing, energy construction | 52 (23.1) | 89 (39.6) | 67 (29.8) |  | 26 (8.9) | 48 (16.5) | 39 (13.4) |  |  | |  | |  |
| Services | 162 (72.0) | 132 (58.7) | 146 (64.9) |  | 258 (88.7) | 237 (81.4) | 242 (83.2) |  |  | |  | |  |
| **Company size** |  |  |  |  |  |  |  |  |  | |  | |  |
| Small-medium (≤ 100 workers) | 129 (57.3) | 135 (60.0) | 153 (68.0) | 0.001** | 158 (54.3) | 143 (49.1) | 170 (58.4) | 0.058 |  | |  | |  |
| Big (>100 workers) | 89 (39.6) | 90 (40.0) | 72 (32.0) |  | 131 (45.0) | 148 (50.9) | 121 (41.6) |  |  | |  | |  |
| **Company ownership** |  |  |  |  |  |  |  |  |  | |  | |  |
| Private | 161 (71.6) | 179 (79.6) | 175 (77.8) | 0.001** | 193 (66.3) | 203 (69.8) | 197 (67.7) | 0.314 |  | |  | |  |
| Public | 44 (19.6) | 29 (12.9) | 28 (12.4) |  | 64 (22.0) | 57 (19.6) | 53 (18.2) |  |  | |  | |  |
| **5 years prior to follow-up** |  |  |  |  |  |  |  |  |  | |  | |  |
| **Employment time ratio (mean (SD))** | 90.9 (20.6) | 93.1 (17.1) | 93.2 (16.1) | 0.019** | 91.8 (17.7) | 92.7 (17.2) | 93.1 (16.1) | 0.278 |  | |  | |  |

SA, sickness absence; Follow-up period ranged from 3 to 7 years from entrance to the cohort until the end of 2018; Previous 5 years refers to each individual´s entrance; SD, standard deviation. *p<0.05, **p<0.01, ***p<0.001.

| *Supplementary table 2: Cancer location in the group of workers with SA due to cancer and diagnosis underlying SAs in the comparison group with other causes by sex in Catalonia (2012-2015)* | | |  |
| --- | --- | --- | --- |
|  |  |  |  |
| **Comparison group** | **Men** | **Women** |  |
| **SA due to cancer diagnosis** | N (%) | N (%) |  |
| Lip, oral cavity, and pharynx | 3 (1.3) | 2 (0.7) |  |
| Digestive organs | 42 (18.7) | 25 (8.6) |  |
| Respiratory system | 19 (8.4) | 6 (2.1) |  |
| Bone and articular cartilage | 1 (0.4) | * |  |
| Skin | 27 (12.0) | 53 (18.2) |  |
| Connective and soft tissue | 3 (1.3) | * |  |
| Breast and female genital organs | 1 (0.4) | 152 (52.2) |  |
| Male genital organs | 52 (23.1) | * |  |
| Urinary organs | 41 (18.2) | 17 (5.8) |  |
| Eye, brain, and central nervous system | 10 (4.4) | 5 (1.7) |  |
| Endocrine glands and related structures | 6 (2.7) | 15 (5.2) |  |
| Secondary and ill-defined | 6 (2.7) | 7 (2.4) |  |
| Lymphoid, haematopoietic, and related tissue | 14 (6.2) | 8 (2.7) |  |
| Multiple locations | * | 1 (0.3) |  |
| **Total** | 225 (100) | 291 (100) |  |
| **SA due to other diagnoses** | N (%) | N (%) |  |
| Infectious and parasitic diseases | 33 (14.7) | 32 (11.0) |  |
| In situ, benign neoplasms | * | 9 (3.1) |  |
| Endocrine, nutritional, and metabolic diseases | * | 2 (0.7) |  |
| Mental and behavioural disorders | 15 (6.7) | 22 (7.6) |  |
| Diseases of the nervous system | 2 (0.9) | 12 (4.1) |  |
| Diseases of the eye and adnexa | 4 (1.8) | 6 (2.1) |  |
| Diseases of the ear and mastoid process | 4 (1.8) | 7 (2.4) |  |
| Diseases of the circulatory system | 10 (4.4) | 12 (4.1) |  |
| Diseases of the respiratory system | 46 (20.4) | 47 (16.2) |  |
| Diseases of the digestive system | 17 (7.6) | 19 (6.5) |  |
| Diseases of the skin and subcutaneous tissue | 2 (0.9) | 7 (2.4) |  |
| Diseases of the musculoskeletal system and connective tissue | 59 (26.2) | 68 (23.4) |  |
| Diseases of the genitourinary system | 10 (4.4) | 10 (3.4) |  |
| Pregnancy, childbirth, and the puerperium | 1 (0.4) | 4 (1.4) |  |
| Symptoms, signs, and abnormal clinical and laboratory findings | 4 (1.8) | 17 (5.8) |  |
| Injury, poisoning, and certain other consequences of external causes | 17 (7.6) | 16 (5.5) |  |
| Factors influencing health status and contact with health services | 1 (0.4) | 1 (0.3) |  |
| **Total** | 225 (100) | 291 (100) |  |
| *N, Absences between 2012 and 2015; SA, sickness absence; MD(P25, P75), median duration and 25th and 75th percentiles of the absences. | | |  |

*Supplementary table 3 Selection of cluster solution and cluster quality measure Average Silhouette Width (ASW) for future working life of a sample of salaried workers in Catalonia (2012-2018) with 8 possible states.*

| **Global ASW** | | **Women** | **Men** |
| --- | --- | --- | --- |
| 3 Clusters | 0.42 | | 0.41 |
| 4 Clusters | 0.45 | | 0.45 |
| **5 Clusters** | **0.47** | | **0.48** |
| Values closer to 1 were strongly well clustered; values closer to 0 denote weak structures that could be artificial. | | | |
